# Probing Dynamics within Amyloid Fibrils Using a Novel Capping Method**

**DOI:** 10.1002/anie.200901343

**Published:** 2009-01-01

**Authors:** Geoffrey W. Platt, Wei-Feng Xue, Steve W. Homans, Sheena E. Radford

**Affiliations:** Astbury Centre for Structural Molecular Biology University of Leeds, Leeds, LS2 9JT (UK)

**Keywords:** diffusion, fibrillar proteins, NMR spectroscopy, proteolysis

A host of diseases involve deposition of proteinaceous amyloid fibrils, which are highly ordered, noncovalent polymers that contain a cross-β architecture. Despite great interest in these fibers, knowledge of the atomic structure of amyloid is limited owing to the difficulty of studying these large heterogeneous biomolecules, especially those formed from long polypeptide chains, with any single biophysical method. Solid-state NMR spectroscopic methods have provided information on the arrangement of the polypeptide chain within amyloid-like structures, affording constraints for secondary, tertiary, and quaternary structure.^[1]^ Herein we study the manner in which the polypeptide chain of β_2_-microglobulin (β_2_m), a 99-residue protein that forms amyloid-like fibrils in vitro and in vivo,^[2,3]^ is accommodated within its fibril architecture. By employing a novel method that decouples the interfering contributions of dynamic exchange between fibrillar and soluble material in structural analyses by solution NMR spectroscopy,^[4]^ we discern which regions of β_2_m are structured in the core of the fibrils, which are exposed, and which are dynamic.

Limited proteolysis of β_2_m fibrils with pepsin has shown that the N-terminal nine residues are exposed to solvent and that digestion of this sample results in a homogeneous product in which 100% of the fibrils are cleaved at a single site (Val9) (Figure 1a and Supporting Information, Figure S1). These data are consistent with NMR spectroscopy hydrogen exchange experiments that reveal limited protection in the 20 N-terminal residues of these fibrils.^[5]^ However, little is known about the dynamics of the polypeptide chain when it is organized into the fibril structure. Recently solid-state NMR spectroscopy methods have identified flexible regions in amyloid fibrils,^[6, 7]^ and previous studies have indicated that mobile regions within large macromolecules can be observed by solution NMR spectroscopy, even though the size of the systems examined would usually prohibit the use of this technique.^[8–10]^

To better understand the structural organization of the polypeptide chain in β_2_m amyloid-like fibrils (Figure 1b) and to identify possible mobile regions within this system, fibril formation of β_2_m was monitored in real time by ^1^H–^15^N HSQC NMR spectroscopy (Figure 2a,b). In parallel, the progression of the fibrillation reaction was monitored by fluorescence of the amyloid-specific dye thioflavin-T as well as by imaging with TEM.^[2]^ Typical thioflavin-T-positive long-straight and twisted amyloid-like fibrils were observed at the conclusion of the reaction (Figure 1b). The initial NMR spectrum (Figure 2a), which was acquired as soon as the protein was placed under low-pH-value conditions, is typical of acid-unfolded β_2_m, in which a number of intense resonances are observed with limited chemical shift dispersion, indicative of a highly unfolded polypeptide chain.^[11]^ As the reaction proceeds, peak intensities throughout the protein sequence are decreased as monomeric protein is recruited to the fibrillar form, leading to broadened contributions to their linewidths. At the endpoint of the reaction (after 250 h), a surprising number of peaks remains visible in the spectrum (Figure 2b). Interestingly, no chemical-shift changes are observed for these resonances on conversion to the fibrillar state, allowing their assignment to residues within the 20 N-terminal amino acids of the sequence. Analysis of the molecular dimensions of the species giving rise to these resonances using NMR spectroscopic diffusion methods^[10, 12]^ revealed that the signals observed result from monomeric β_2_m with diffusion profiles consistent with the acid-unfolded monomers present prior to assembly (Figure 3). These species most likely represent monomers in equilibrium with the assembled form. Indeed, molecular recycling from the ends of amyloid fibrils has been reported for fibrils created from other proteins,^[4]^ potentially giving rise to complexities in the structural interpretation of experimental data. Alternatively, it is possible that unpolymerized monomers give rise to the signals observed.

To rule out contributions from subunit exchange and residual monomers to the observed NMR spectroscopy signal of β_2_m fibrils, a method was developed based upon one of the fundamental characteristics of amyloid that arises from its nucleated assembly mechanism—the ability to seed. In this method, uniformly ^15^N-labeled β_2_m (178 μm) was used to form fibrils. The fibrils were then pelleted by centrifugation and resuspended in buffer containing high concentrations (356 μm) of monomeric ^14^N-β_2_m. This procedure allows the ^15^N-labeled fibrils to be rapidly elongated,^[2,3]^ creating ^15^N-labeled fibrils containing ^14^N-β_2_m “caps”. As well as removing the possibility that monomer exchange from the fibril ends will contribute to the ^1^H–^15^N HSQC spectrum,^[4]^ the addition of the ^14^N-labeled monomer also significantly decreases the amount of ^15^N-labeled β_2_m that remains in monomeric form at the end of the assembly reaction. The ^1^H–^15^N HSQC spectrum of capped fibrils created in this manner is bereft of peaks (Figure 2c), thus confirming that the resonances observed in the spectrum of the sample that lacks fibril capping arise from monomeric protein. That the observable resonances in the spectrum of uncapped fibrils correspond chiefly to N-terminal residues can be explained by the fact that these give the most intense peaks in the spectrum of the acid-unfolded monomer.^[11]^ Importantly, these experiments demonstrate a complete lack of observable resonances in the backbone of the β_2_m polypeptide chain in the fibrillar state, despite the fact that the N-terminal segment is accessible to protease cleavage (Supporting Information, Figure S1) and is relatively poorly protected from hydrogen exchange.^[5]^

We exploited the capping method to assess the structure and dynamics in the N-terminal of fibrillar β_2_m by creating a variant with an elongated sequence comprising six glycine and three serine residues inserted N-terminal of Ile1 (Figure 1a). Fibril growth from this variant, accelerated by seeding with ^14^N-wild-type (WT) β_2_m fibril seeds at low pH values, resulted in fibrils indistinguishable from those formed by the WT protein as imaged by TEM (Figure 1c). As with the fibrils formed from the WT protein, the N-terminal region of the extended variant was also shown to be specifically sensitive to pepsinolysis at Val9 (WT numbering), consistent with the variant adopting a similar fibrillar architecture to its WT counterpart (data not shown). Analysis of fibril assembly of this variant by NMR spectroscopy revealed that the monomeric form of the protein is unfolded at pH 2.5 (Figure 2d) and that resonances belonging to the N-terminal extended region, as well as the natural approximately 20 N-terminal residues, of the protein are visible at the endpoint of fibril growth (Figure 2e). However, in marked contrast to the WT sample, NMR spectroscopic diffusion measurements showed that the observed signals in the NMR spectrum originate predominantly from species much larger than monomer (more than 10-times larger, Figure 3), demonstrating that the obtained values for the uncapped fibrils are average contributions from of both fibrillar species and residual monomer. Furthermore, resonances corresponding to the extended N-terminal region remain visible in the ^1^H–^15^N HSQC spectrum of the ^14^N-β_2_m capped fibrils (Figure 2f), indicating that this region of the polypeptide chain displays dynamics on the nanosecond to picosecond timescale, independent of the fibrils. Consistent with this analysis, the N-terminal residues exhibit chemical shifts identical to those of the acid-unfolded monomer, and their increased linewidths reflect the reduced overall correlation time of the fibrils. These results confirm the ability to detect dynamic regions within amyloid fibrils by solution NMR spectroscopy, provided that care is taken to remove artifacts arising from subunit cycling or residual monomer.

Current insights into the architecture of β_2_m fibrils suggest models in which residues 10–99 are arranged in a highly protected cross-β core involving parallel β strands that are constrained by the persistence of the disulfide bond linking cysteine residues 25 and 80.^[13, 14]^ While the 10–20 N-terminal residues of β_2_m in the fibrillar form are susceptible to both proteolysis and relatively rapid hydrogen exchange,^[5, 15]^ the data presented indicate that this region of the protein does not display mobility independent of the fibril core but gives rise to resonances broadened beyond detection using NMR spectroscopy. We therefore propose that while Val9 is exposed in the amyloid-like state of β_2_m, substantial and stable interactions must exist between approximately the 20 N-terminal residues and the rest of the fibril core such that this region of the polypeptide chain does not display dynamics, at least on the nanosecond to picosecond timescale, independent of the remainder of the fibril. Addition of nine residues propagates the structure of this region into solution, implying that the first residues of WT β_2_m are oriented outwards from the fibril core. The evidence provided indicates that while the N-terminal region of β_2_m is not highly protected in the fibril core, it is nonetheless integral to the fibril core architecture, a feature that must be considered when developing structural models of these fibrils.

The work described herein demonstrates the important role that solution NMR spectroscopy can play in deciphering the properties of dynamic regions of amyloid fibrils and reveals new information about the β_2_m amyloid core. The method utilized for reducing the effect of molecular recycling or the presence of residual monomer in solution on NMR spectroscopy studies of amyloid-like fibrils is generally applicable, ensuring the molecular origins of signals in NMR spectra and providing a powerful technique for examining the structural features of these important macro-molecular assemblies.

## Supplementary Material

Supporting information for this article, including experimental details, is available on the WWW under http://dx.doi.org/10.1002/anie.200901343.

Supporting Information

## Figures and Tables

**Figure 1:**
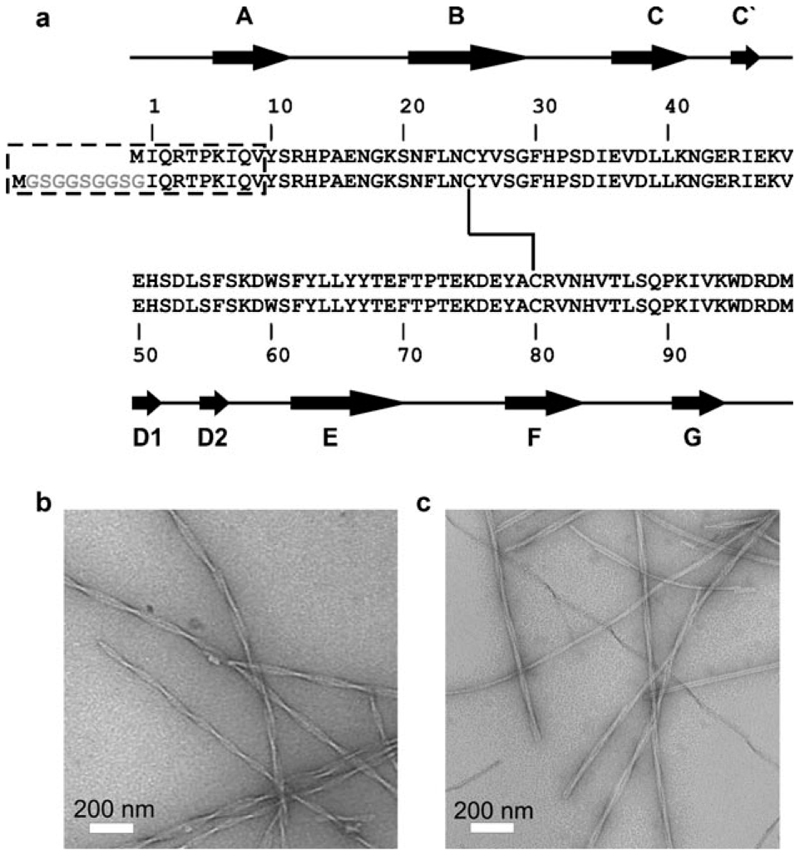
a) Sequence of wild-type (WT) β_2_m and the variant with an extended N-terminal sequence. The fragments prone to pepsinolysis are highlighted within the dashed box and positions of secondary structure and the disulfide bond in the native state are indicated. b, c) Negative-stain TEM images of fibrils formed at pH 2.5 from WT β_2_m (b) and N-terminally extended β_2_m (c).

**Figure 2:**
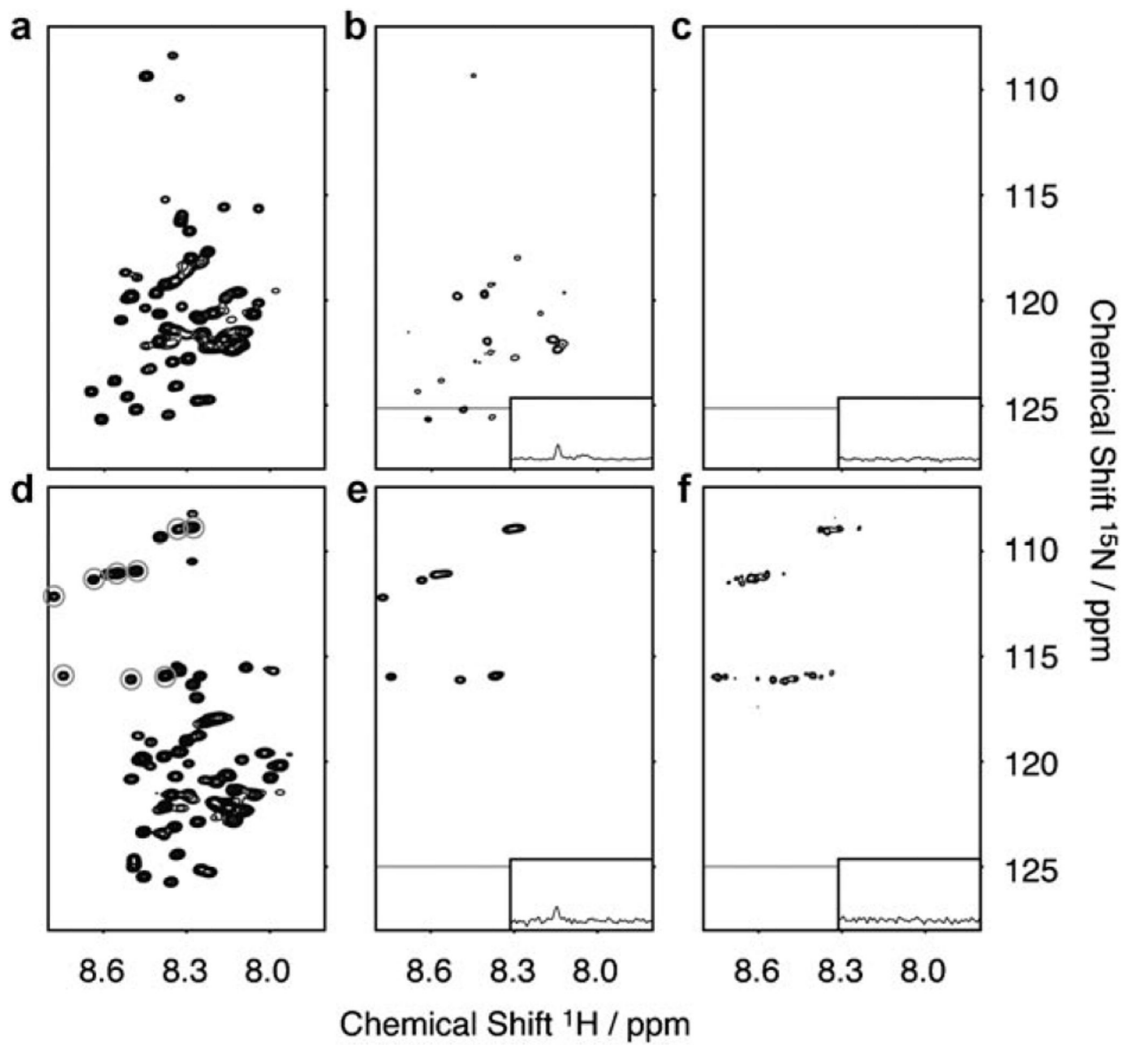
a–c) WT β_2_m fibril growth at pH 2.5 and 25 °C monitored by ^1^H–^15^N HSQC NMR spectroscopy at a) *t* = 0 and b) *t* = 250 h (at the endpoint of the reaction); c) endpoint after fibril capping with ^14^N β_2_m. d–f) ^1^H–^15^N HSQC NMR spectra of the seeded fibril growth of the N-terminal extension variant of β_2_m at d) *t*=0, e) *t*=250 h, and f) after capping with ^14^N-labeled WT monomers. Resonances corresponding to the extended sequence are ringed in (d), and other resonances were previously assigned.^[11]^ Contours of (b), (c), and (f) are set eight times lower than (a), (d), and (e) owing to lower signal intensity. 1D slices at the ^15^N frequencies marked by a gray horizontal line are shown in the insets of (b), (c), (e), and (f). These slices illustrate the detectable presence of residual monomeric β_2_m in the uncapped fibrillar sample (see also Supporting Information Figure S2).

**Figure 3:**
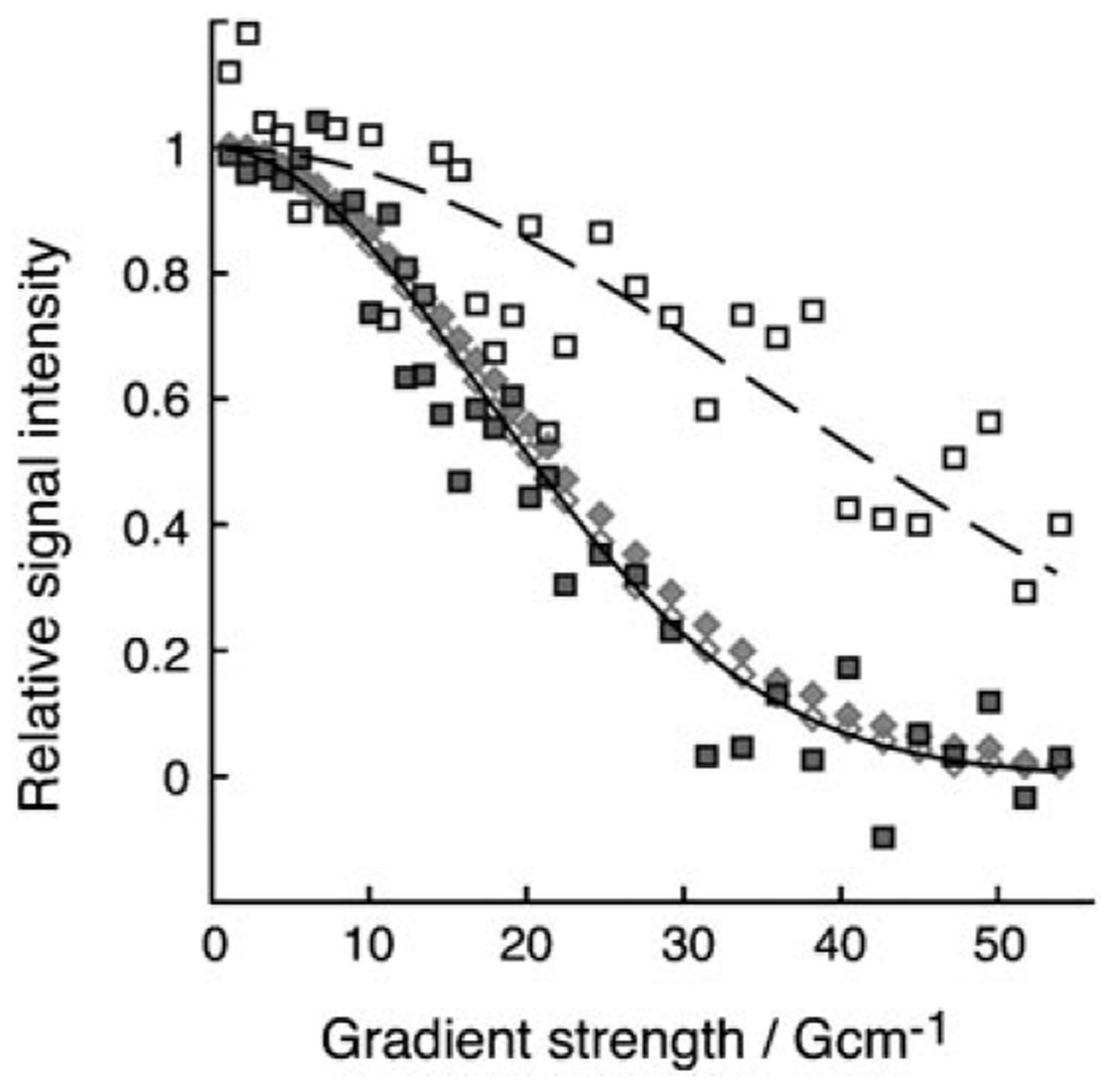
DOSY signal decay profile for monomeric (gray diamonds) and fibrillar (black squares) samples of β_2_m measured from the methyl and NH region of 1D ^1^H NMR spectra of WT β_2_m (filled) and β_2_m with the extended N-terminal sequence (open). The solid and dashed lines represent fits (see the Supporting Information) to the open symbols for the monomeric and uncapped fibrillar samples, respectively.
