# Supplementary material for: Probing Dynamics within Amyloid Fibrils Using a Novel Capping Method**
Source: Angew Chem Int Ed Engl. Author manuscript; Available in PMC 2025 May 28. (PMC7617714; doi:10.1002/anie.200901343)
Supplement: Supporting Information [file EMS205753-supplement-Supporting_Information.pdf]

Angewandte Chemie
Eine Zeitschrift der Gesellschaft Deutscher Chemiker

Supporting Information

© Wiley-VCH 2009

69451 Weinheim, Germany

Probing Dynamics within Amyloid Fibrils Using a Novel Capping Method

Geoffrey W. Platt, Wei-Feng Xue, Steve W. Homans & Sheena E. Radford.

Supporting Information

S1- Methods

Protein Purification and Fibril Growth

Human β_2-microglobulin (β_2m) was expressed and purified as described elsewhere. [1] Fibrils were formed in 25 mM sodium acetate/ 25 mM sodium phosphate buffer at pH 2.5 with agitation (200 rpm) and purified by centrifugation at 16, 300 x *g* for 30 min, as described previously. [2, 3]

Limited Proteolysis

Purified fibrils were resuspended in 25 mM sodium acetate / 25 mM phosphate buffer at pH 2.5 and treated with pepsin (Sigma), a general endopeptidase that has been previously shown to cleave more than thirty sites throughout the sequence of unfolded β_2m under the conditions employed. [4] The fibrils were treated for 1.5 h at 37 °C with 100 μg pepsin added per 1 mg of fibrillar β_2m. The digestion was terminated by addition of pepstatin (Sigma, 10 μM final concentration). The reaction was studied by SDS-PAGE analysis using a 15 % (*w/v*) polyacrylamide gel, by ESI-MS and by ^{1}H-^{15}N HSQC spectra.

TEM

Bright field TEM images were taken on a Philips CM10 transmission microscope operating at 80 kV using fibrils stained with 4 % (*w/v*) uranyl acetate.

NMR

NMR data were acquired on Varian Unity Inova spectrometers operating at proton frequencies of 500 and 600 MHz. Gradient-enhanced ^{1}H-^{15}N HSQC spectra were acquired using 128 complex points and 16 scans per increment. Watergate solvent suppression was used and all NMR data were processed using NMRPipe. [5] The diffusion ordered spectroscopy (DOSY) experiments were carried out using the BPPSTE pulse sequence. [6] The integrated signal *I* as function of gradient strength *g* was fitted to a Gaussian decay with amplitude *A* and decay constant *d*:

$$I = A\,exp(dg^2)$$

Figure 3 (main text) shows the relative signal intensity (*I/A*) measured from the methyl and NH region of 1D ^{1}H NMR spectra *versus* gradient strength (*g*). The relative change in diffusion constant of the fibrillar samples compared with monomer diffusion was then characterised by the relative change in the decay constant *d*.

S2 – Limited Proteolysis Data

The limited proteolysis experiments were designed to confirm which regions of β_2m are protected in the core of the fibrillar state. The experiments were performed as described above. SDS-PAGE analysis (Figure S1a) indicates that under these conditions monomeric β_2m is digested completely into peptides that are too small to be observed on a 15 % (*w/v*)

polyacrylamide gel, in agreement with previous findings studied by ESI-MS(/MS). [4] The fibrils are, however, only partially cleaved by the enzyme and a large portion of the constituent ~ 12 kDa β_2m polypeptide sequence is protected from digestion, as indicated by a band reduced by approximately 1.4 kDa on the gel. To define which part of the β_2m sequence is protected from pepsinolysis, the fibrils were depolymerised before and after digestion and the resulting monomers analysed by NMR and mass spectrometry. Depolymerisation was achieved using the following method: after suspension of the digestion reaction the fibril samples were centrifuged, the pellets washed in 5 mM HCl and then lyophilised. The lyophilised samples were resuspended in a 99 % (*v/v*) DMSO-d_6, 1 % (*v/v*) H_2O solution, similar to that described for whole-fibril hydrogen exchange of β_2m aggregates. [7] This buffer allows rapid dissolution of the fibrils into a DMSO-unfolded monomeric state that is amenable to NMR. [8] ^{1}H-^{15}N HSQC NMR spectra were acquired after a 15-minute dead time to allow for experimental set-up. Comparison of the spectra of the fibrils that were partially digested with those that were treated identically, but without the addition of pepsin, (Figure S1b) shows that a number of resonances, mainly those corresponding to N-terminal 12 residues are removed, according to published assignments. [9] Mass spectrometric analysis of the DMSO-denatured fibrils revealed the presence of only a single fragment corresponding to β_2m lacking the nine N-terminal residues and the release of a single N-terminal nine-residue fragment that is not cleaved further by pepsin (data not shown).

Supplementary Figure Legends

Figure S1. a) 15 % (*w/v*) SDS-PAGE of monomeric and fibrillar β_2m with (+) and without (-) pepsin treatment after 1.5 h. ***b)*** ^{1}H-^{15}N HSQC spectra of 178 μM β_2m fibrils dissolved in 100 % DMSO-d_6 before (black) and after (red) treatment with pepsin and purification by centrifugation. Assigned resonances from the N-terminal region are labelled. The spectra were acquired at 25 °C using a spectrometer operating at a ^{1}H frequency of 600 MHz. * unassigned resonance.

Figure S2. 1D slices of ^{1}H-^{15}N HSQC spectra of 'uncapped' fibrils shown in Figure 2. ***a)*** 1D slice of spectrum of WT β_2m fibrils (Figure 2b) at ^{15}N shift of 125.0 ppm. ***b)*** 1D slice of WT β_2m fibrils at ^{15}N shift of 115.9 ppm. ***c)*** 1D slice of fibrils grown from the N-terminal extension variant of β_2m (Figure 2e) at ^{15}N shift of 125.0 ppm. ***d)*** 1D slice of fibrils grown from the N-terminal extension variant of β_2m at ^{15}N shift of 115.9 ppm.

References

[1] N. M. Kad, N. H. Thomson, D. P. Smith, D. A. Smith, S. E. Radford, *J. Mol. Biol.* **2001**, *313*, 559.
[2] G. W. Platt, K. E. Routledge, S. W. Homans, S. E. Radford, *J. Mol. Biol.* **2008**, *378*, 251.
[3] W. F. Xue, S. W. Homans, S. E. Radford, *Proc. Natl. Acad. Sci. U. S. A.* **2008**, *105*, 8926.
[4] S. L. Myers, N. H. Thomson, S. E. Radford, A. E. Ashcroft, *Rapid Commun. Mass Spectrom.* **2006**, *20*, 1628.
[5] F. Delaglio, S. Grzesiek, G. W. Vuister, G. Zhu, J. Pfeifer, A. Bax, *J. Biomol. NMR* **1995**, *6*, 277.
[6] M. D. Pelta, H. Barjat, G. A. Morris, A. L. Davies, S. J. Hammond, *Magn. Reson. Chem.* **1998**, *36*, 706.
[7] M. Hoshino, H. Katou, Y. Hagihara, K. Hasegawa, H. Naiki, Y. Goto, *Nat. Struct. Biol.* **2002**, *9*, 332.
[8] N. Hirota-Nakaoka, K. Hasegawa, H. Naiki, Y. Goto, *J. Biochem.* **2003**, *134*, 159.
[9] K. I. Yamaguchi, H. Katou, M. Hoshino, K. Hasegawa, H. Naiki, Y. Goto, *J. Mol. Biol.* **2004**, *338*, 559.

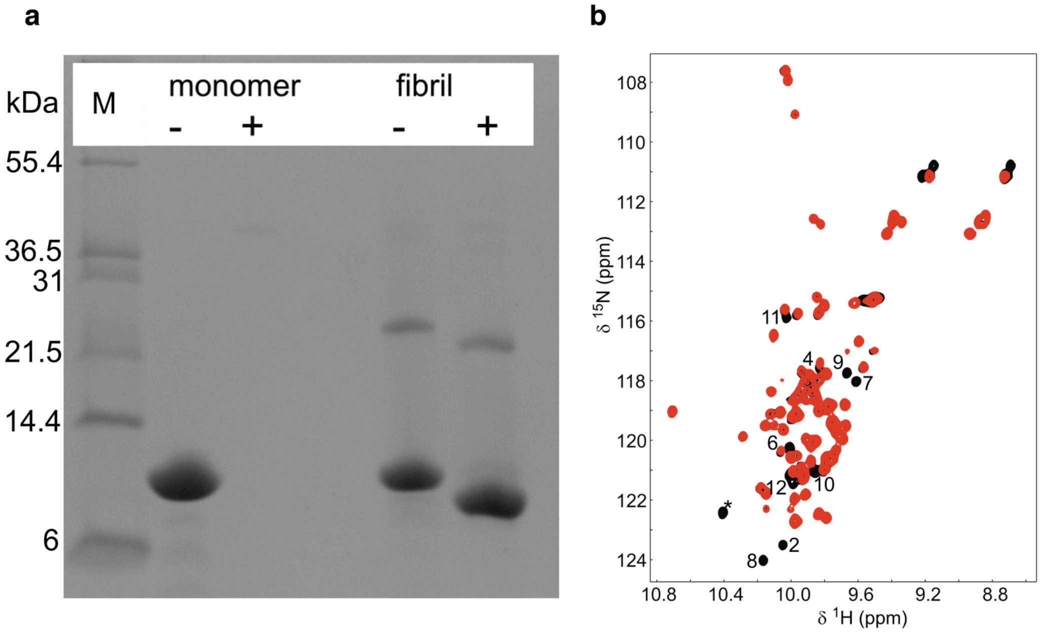

Figure S1

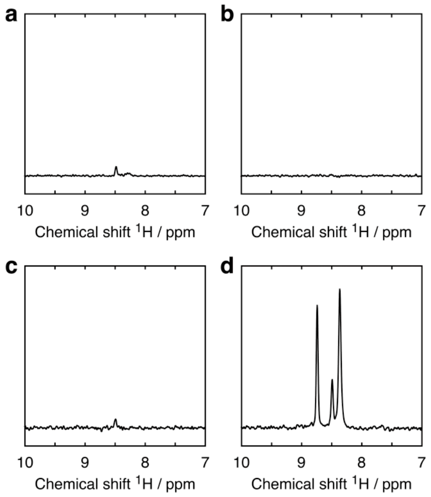

Figure S2
